# Membrane Fouling Potentials of an Exoelectrogenic Fouling-Causing Bacterium Cultured With Different External Electron Acceptors

**DOI:** 10.3389/fmicb.2018.03284

**Published:** 2019-01-14

**Authors:** So Ishizaki, Rimana Islam Papry, Hiroshi Miyake, Yuko Narita, Satoshi Okabe

**Affiliations:** Division of Environmental Engineering, Faculty of Engineering, Hokkaido University, Sapporo, Japan

**Keywords:** integrated MFC and MBR system, membrane fouling, exoelectrogenic fouling-causing bacteria (FCB), anodic respiration, biopolymer

## Abstract

Integrated microbial fuel cell (MFC) and membrane bioreactor (MBR) systems are a promising cost-effective and energy-saving technology for wastewater treatment. Membrane fouling is still an important issue of such integrated systems in which aeration (oxygen) is replaced with anode electrodes (anodic respiration). Here, we investigated the effect of culture conditions on the membrane fouling potential of fouling-causing bacteria (FCB). In the present study, *Klebsiella quasipneumoniae* strain S05, which is an exoelectrogenic FCB isolated from a MBR treating municipal wastewater, was cultured with different external electron acceptors (oxygen, nitrate, and solid-state anode electrode). As results, the fouling potential of S05 was lowest when cultured with anode electrode and highest without any external electron acceptor (*p* < 0.05, respectively). The composition of soluble microbial products (SMP) and extracellular polymeric substances (EPS) was also dependent on the type of electron acceptor. Protein and biopolymer contents in SMP were highly correlated with the fouling potential (*R*^2^ = 0.73 and 0.81, respectively). Both the fouling potential and yield of protein and biopolymer production were significantly mitigated by supplying electron acceptors sufficiently regardless of its types. Taken together, the aeration of MBR could be replaced with solid-state anode electrodes without enhancement of membrane fouling, and the anode electrodes must be placed sufficiently to prevent the dead spaces in the integrated reactor.

## Introduction

It is particularly important to reduce or omit energy intensive aeration in order to facilitate wider applications of membrane bioreactors (MBRs) (Ren et al., 2014; Yuan and He, 2015; Li et al., 2016). In this regards, integration of microbial fuel cell (MFC) and MBR is an interesting approach, because the integrated system has several advantages, including better effluent water quality, no or less requirement of aeration, and reduction of sludge (Wang et al., 2011; Yuan and He, 2015; Katuri et al., 2018). However, there are still serious concerns about severe membrane fouling if aeration is reduced or omitted in the integrated system (Malaeb et al., 2013a; Ren et al., 2014).

It has been recently reported that membrane fouling was mitigated by integrating MFC and MBR due to less production of soluble microbial products (SMP) and extracellular polymeric substances (EPS) under anodic respiration conditions (Su et al., 2013; Tian et al., 2014; Ma et al., 2015). In particular, the production of biopolymer, which is well known as a main foulant, was significantly reduced in mixed population biomass in electrode-associated MBR (e-MBR) (Tian et al., 2013, 2014; Ishizaki et al., 2016b). Furthermore, a pure culture of *Geobacter sulfurreducens* strain principle compartment analysis (PCA), an exoelectrogenic bacterium, produced less biopolymer and consequently exhibited lower membrane fouling under anodic respiration condition than under fumarate (anaerobic) respiration condition (Ishizaki et al., 2016b).

In our previous studies, 41 bacterial strains that displayed significant fouling potential [herein defined as fouling-causing bacteria (FCB)] were isolated from MBR treating municipal wastewater, and their membrane fouling potentials were determined when cultured as single-culture and co-culture (Ishizaki et al., 2016a, 2017). Among the isolated FCB, we found phylogenetically diverse exoelectrogens, which have the ability to respire with solid-state anode electrode as a terminal electron acceptor (Logan, 2009; Ishizaki et al., 2016a). However, since there were no anodes in the MBR where the exoelectrogenic FCB were isolated from, they might have used other external electron acceptors such as oxygen and nitrate. In the case of the integrated MFC and MBR system, these exoelectrogenic FCB could use anode electrodes, which might influence the membrane fouling. However, there is no information on the effect of different electron acceptors on the fouling potential of exoelectrogenic FCB.

Therefore, we investigated the effect of different electron acceptors (i.e., solid-state anode electrode, oxygen, and nitrate) on the production of SMP and EPS including biopolymer by an exoelectrogenic FCB and consequent membrane fouling, because SMP was a primarily contributor for membrane fouling occurring in e-MBR as like conventional MBR (Meng et al., 2009; Guo et al., 2012; Ishizaki et al., 2016b). *Klebsiella quasipneumoniae* strain S05 (Ishizaki et al., 2016a; Kitajima et al., 2018) was selected from the isolated FCB, because this strain exhibited severe membrane fouling and has the ability to use solid-state anode (anode respiration), oxygen, and nitrate as a terminal electron acceptor (Ishizaki et al., 2016a). In addition, this strain can ferment glucose. We report here that S05 yielded the lowest fouling potential when cultured with solid-state anode electrode applied an electrical potential (+0.2 V vs. Ag/AgCl) and the highest fouling potential when cultured without any external electron acceptor (i.e., fermentation condition).

## Materials and Methods

### Bacterial Strain

*Klebsiella quasipneumoniae* strain S05, which is closely related to *K. pneumoniae* (99.5% 16S rRNA gene similarity), was previously isolated from a pilot-scale MBR treating real domestic wastewater (Ishizaki et al., 2016a). In our previous study, totally 15 isolates were characterized as FCB, and 3 out of 15 isolates (Strain S05, S32, and S33) formed colony on R2A agar plate containing 20 mM of ferric citrate under anaerobic condition (Chung and Okabe, 2009a). In this study, S32 and S33, shared ≥99.9% 16S rRNA gene sequence identity and affiliated with *Paenibacillus polymyxa*, were not used, because this family was very minor in e-MBR in our previous study (Ishizaki et al., 2016a,b). Strain S05 displayed about 10 times higher fouling potential as compared with the other isolated strains and was regarded as FCB (Ishizaki et al., 2017). *K. pneumoniae* is a facultative anaerobe and known to generate high electricity from glucose and starch with self-producing electron shuttles (Zhang et al., 2008; Kumar et al., 2015).

### Reactor Configuration and Operational Condition

To examine the effect of different respiration modes on membrane fouling potential, strain S05 was cultured with different electron acceptors (solid-state anode electrode, oxygen, nitrate, and none) in double-chamber MFCs. The double-chamber MFC consisted of an anode chamber (250 ml) and a cathode chamber (250 ml) (Supplementary Figure S1) (Ishizaki et al., 2016b). The porous carbon (6 cm × 5 cm, Somerset; NJ, United States) and carbon cloth loaded with 0.5 mg/cm^2^ of platinum (3 cm × 5 cm, E-TEK, Somerset; NJ, United States) were used as an anode electrode and a cathode electrode, respectively (Ishizaki et al., 2014). Each chamber was separated with a Nafion membrane (Nafion^TM^ 117, Dupont Co., DE, United States). Anodic respiration was facilitated by applying an electrical potential to the anode (+0.2 V vs. Ag/AgCl) using a potentiostat/galvanostat (HA-151B, Hokuto Denko Co., Tokyo, Japan), which was defined as sufficient supply (Supplementary Figure S1). Anodic respiration without applied electrical potential was defined as short supply. For the short supply, the double-chamber MFC was operated with external resistance of 1 ohm (Supplementary Figure S1). To confirm that abiotic reaction did not mitigate the membrane fouling, heat-sterilized SMP was incubated in a close-circuit MFC applied electrical potential to the anode (+0.2 V vs. Ag/AgCl) and an open-circuit MFC, and then membrane resistance was monitored for 2 days as described elsewhere.

For oxygen and nitrate respiration, the double-chamber MFC was operated with open electric circuit (Supplementary Figure S1). Each electron acceptor was supplied at two different levels; sufficient supply and short supply. For sufficient oxygen and nitrate supply studies, ambient air was continuously fed at a flow rate of 25 L/h and 2 ml of 6 M NaNO_3_ solution was supplemented every day, respectively. Dissolved oxygen (DO) concentration and nitrate concentration were monitored to ensure no limitation by using a DO meter (DO-5Z; Kasahara Chemical Instruments Co.; Saitama, Japan) and an ion-exchange chromatography (IC-2010, TOSOH; Tokyo, Japan), respectively. For short supply studies, 2% O_2_ gas (pure N_2_/Air = 4:1) was continuously fed at a flow rate of 0.45 ml/min and 1 ml of 0.6 M NaNO_3_ solution was supplemented every day, respectively. Open circuit MFC was also operated without any dissolved external electron acceptor as a control (none).

After overnight preincubation, the S05 culture was washed twice with and incubated into a modified M9 medium with the following composition: 200 μM (NH_4_)_2_SO_4_, 200 μM NaCl, 500 μM CaCl_2_, 500 μM MgCl_2_⋅6H_2_O, 27 mM K_2_HPO_4_, 55 mM KH_2_PO_4_ and 20 mM glucose (the sole energy source). The initial biomass concentration was adjusted at OD_600_ = 0.5. Each reactor was operated for 2 or 4 days as a batch mode at room temperature (25 ± 2°C).

### Bacterial Growth

Time variation in the biomass concentration (OD_600_) in each reactor was monitored for 2 or 4 days. The initial biomass concentration was adjusted at OD_600_ = 0.1. The OD_600_ value was measured by using an optical absorbance meter (Smart Spec Plus; Bio-Rad; CA, United States). To quantify the bacterial abundance in mixed liquor (ML) and on anode electrode as based on the number of 16S rRNA gene, qPCR assay was performed using SYBR Green chemistry as described previously with small modification (Kobayashi et al., 2013; Ishizaki et al., 2016b). DNA was extracted from ML (1 ml) and anode electrode (ca. 5 mm × 5 mm) using a Fast DNA Spin kit for soil (Bio101, Vista; CA, United States). In SYBR Green assays, each PCR mixture (10 μl) was composed of 1× SYBR Premix Ex Taq II (Takara Bio, Otsu, Japan), 50× ROX Reference Dye (Takara Bio, Otsu, Japan), 400 nM each of forward and reverse primers, and 2 μl of template DNA. The set of primers of Eub338f (5′-CCTACGGGAGGCAGCAG-3′) and Eub518r (5′-GWATTACCGCGGCKGCTG-3′) were used (Elshahed et al., 2008). COD concentration was measured according to the HACH COD method 8000 by using a HACH COD reactor and spectrophotometer DR/2400 (HACH Co.; Loveland, CO, United States). The H_2_ concentration in the head space of anode chamber was measured by using Gas Chromatography (GC-14B; Shimadzu Co., Kyoto, Japan).

### Measurement of Membrane Fouling Potential

Dead-end filtration test was performed to evaluate the fouling potential as described elsewhere with minor modification (Kimura et al., 2012). After extracting SMP as described below, 5 ml of the SMP solution was transferred to a stirred filtration unit (UHP-25K; Advantec Toyo; Tokyo, Japan) with a flat membrane filter (0.2 μm, hydrophilic PTFE; Advantec Toyo; Tokyo, Japan), and filtered under 50 kPa of ambient air. Thereafter, MilliQ water (10 mL) was added in the filtration unit and filtered again under the same pressure. The permeate flow rate of MilliQ water was measured, and the membrane resistance of the fouled membrane was calculated as follows;

$$\text{Membraneresistance}({\text{m}}^{-}{}^{1})\;\;=\;PA/\text{μ}Q$$

where, *P* is the pressure (Pa), *A* is the filtration area of membrane (m^2^), μ is the viscosity coefficient of MilliQ water (Pa⋅s), and *Q* is the permeate flow rate of MilliQ water (m^3^/s). The membrane resistance was used as a quantitative indicator of membrane fouling potential in this study.

### Extraction and Characterization of SMP and EPS

Soluble microbial products and EPS in bacterial cultures were extracted as described previously with minor modifications (Ramesh et al., 2007; Wang et al., 2009; Ishizaki et al., 2016b). Briefly, 20 ml of bacterial culture was centrifuged at 4°C and 6,000 × *g* for 15 min, and the supernatant was regarded as SMP. The remained biomass pellet was re-suspended in 0.05% NaCl solution (10 mL) and then was subjected to heat treatment at 80°C for 1 h. After dispersed well by vortexing, the suspension was centrifuged again at 4°C and 6,000 × *g* for 15 min. The supernatant was regarded as EPS. Concentrations of TOC, carbohydrate, protein, and biopolymer in the SMP and EPS fractions were measured as described below.

The TOC concentration was measured using a TOC analyzer (TOC-V CSH; Shimadzu; Kyoto, Japan). Carbohydrate and protein concentrations were measured with the phenol-sulfonic acid method with glucose as the standard and the Lowry method with BSA as the standard, respectively. Biopolymer concentration was determined by using Liquid Chromatography with Organic Carbon Detection (LC-OCD Model 8, DOC-LABOR; Karlsruhe, Germany). SMP was also characterized by PCA on the basis of Fourier Transform Infrared (FTIR) spectrum as described in previous studies (Lammers et al., 2009; Ishizaki et al., 2017). The spectrum of the freeze-dried SMP was measured by a FTIR spectrometry (FT/IR-660 Plus; JASCO Co.; Tokyo, Japan). The operating range was from 4,000 to 600 cm^-1^ with a resolution of 10 cm^-1^. PCA was carried out with the normalized spectra by using R 3.0.2 (R Development Core Team; Vienna, Austria).

### Scanning Electron Microscopy

To visualize the production of EPS by S05 under different respiration conditions, bacterial cells were observed by using scanning electron microscopy (SEM) as described in a previous paper with minor modification (Chung and Okabe, 2009b). Bacterial culture was centrifuged at 4°C and 6,000 × *g* for 15 min, the supernatant was discarded, and the pellet was suspended with 0.05% NaCl solution. A small amount of the suspension was placed on the glass plate coated with poly-L-lysine and immersed in 2% (v/v) glutaraldehyde in 0.1 M phosphate buffer for 3 h. The samples were then washed twice with 0.1 M phosphate buffer. The samples were fixed in 1% OsO_4_ in 0.1 M phosphate buffer for 1.5 h and then washed again in the same way. The fixed samples were dehydrated in ethanol series (sequentially in 50, 70, 80, 90, 95, 100, and 100% ethanol for 15 min each) and substituted with isoamyl acetate. The fixed samples were dried with liquid CO_2_ by using a critical-point drier (EM CPD300; Leica Microsystems; Vienna, Austria) and coated with gold and palladium for 2 min (E1030; Hitachi; Tokyo, Japan) at 100 V and 15 mA. The coated samples were examined with an SEM (JSM-6301; JEOL; Tokyo, Japan) at 5 kV.

## Results

### Bacterial Growth

**Figure 1:**
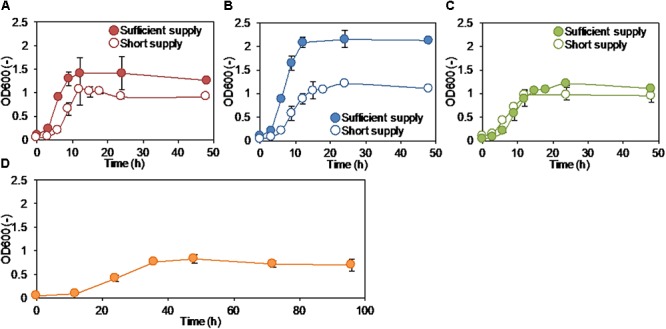
Growth curves of strain S05 when cultured with **(A)** anode electrode, **(B)** oxygen, and **(C)** nitrate as a sole external electron acceptor, respectively. The culture condition with anode electrode regulated the potential at +0.2 V (vs. Ag/AgCl), ambient air at a flow rate of 25 L/h, and 2 ml of 6 M NaNO_3_ solution per a day were categorized as sufficient supply, while those with electrode not regulated the potential, 2% O_2_ gas (pure N_2_/Air = 4:1) at a flow rate of 0.45 ml/min, and 1 ml of 0.6 M NaNO_3_ solution per a day were categorized as short supply, respectively. The growth curve without any electron acceptor was described in **(D)**.

Growth of strain S05 was confirmed in the modified M9 medium with anode electrode, oxygen, and nitrate as the sole external electron acceptor (Figure 1). More than 98% of S05 grew as planktonic cells in the ML rather than biofilms attached on the anode electrode regardless of the type of external electron acceptor (Supplementary Figure S2). Each electron acceptor was supplied at two different levels: sufficient and short supply conditions. Under anode respiration condition, the growth was enhanced by applying an electrical potential to anode (+0.2 V vs. Ag/AgCl) (Figure 1A), which was supported by the increased current generation from 0.21 ± 0.02 to 0.70 ± 0.09 A/m^2^ (Table 1 and Supplementary Figure S3). The highest growth was observed under sufficient oxygen supply condition (Figure 1B). S05 could grow in the modified M9 medium without any external electron acceptor with a significant H_2_ production, suggesting the occurrence of fermentation (Figure 1D and Table 1).

**Table 1:** Performance of S05 cultured with different external electron acceptors.

Electron acceptor	Sufficient supply			Short supply			None
	Electrode	Oxygen	Nitrate	Electrode	Oxygen	Nitrate	
COD consumption (mg/L)	1694 ± 208	3768 ± 34	3648 ± 16	1628 ± 46	1640 ± 292	1896 ± 322	1508 ± 244
Electrical current (mA)	2.11 ± 0.26	–	–	0.64 ± 0.07	–	–	–
Hydrogen production (rnmol)	0.58	NA	ND	2.38	NA	2.94	9.8
Nitrate consumption (mmol)	–	–	8.82 ± 2.22	–	–	NA	–
Coulomb efficiency (%)	8.9 ± 0.6	–	–	2.8 ± 0.3	–	–	–

*NA, not analyzed; ND, not detected*.

H_2_ production was observed from some culture conditions except for sufficient supply of oxygen and nitrate, suggesting that fermentation occurred to some extent (Table 1). This indicates that dissolved external electron acceptors were limited under short supply conditions. For anode respiration, anodic reaction was also limited under both cases with and without the applied electrical potential. Accordingly, more glucose was oxidized (indicated as COD consumption) when more electron acceptors were supplied (Table 1). The lowest COD consumption was observed when cultured without any external electron acceptor (i.e., none).

### Membrane Fouling Potential

**Figure 2:**
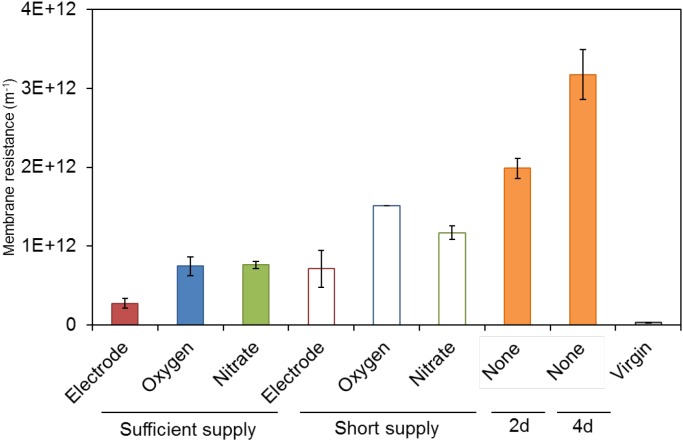
Fouling potential of strain S05 when cultured with different external electron acceptors. Statistical differences were estimated with *P*-value on the basis of two-sample *t*-test, which was summarized in Supplementary Table S1.

The membrane fouling potential of S05 cultured with different electron acceptors was examined by dead-end filtration (Figure 2). Surprisingly, the fouling potential was lowest among the all culture conditions when S05 was cultured with anode electrode with applied potential (*p* < 0.05 except for electrode at short supply) (Figure 2). Although the heat-sterilized SMP of S05 was incubated in a closed-circuit MFC with applied electrical potential for 2 days, the fouling potential remained unchanged (Supplementary Figure S4). This indicates that abiotic effects (such as enhancement of biomass coagulation by electrostatic force) of applied electrical potential to anode (+0.2 V vs. Ag/AgCl) on the membrane resistance was negligible. In contrast, the fouling potential was highest when cultured without any external electron acceptor (none) (*p* < 0.05) even though the biomass concentration (OD_600_) was comparable (Figure 1) and the least COD was oxidized (Table 1). Particularly, the fouling potentials under sufficient supply conditions were significantly lower than without any external electron acceptor (none) (*p* < 0.01) (Supplementary Table S1). Similarly, S32 also caused severe membrane fouling when cultured without any external electron acceptor (*p* < 0.05) (Supplementary Figure S5). In addition, the fouling potential significantly decreased when more oxygen and nitrate were supplied (*p* < 0.05) (Figure 2).

### Characterization in SMP and EPS

**Figure 3:**
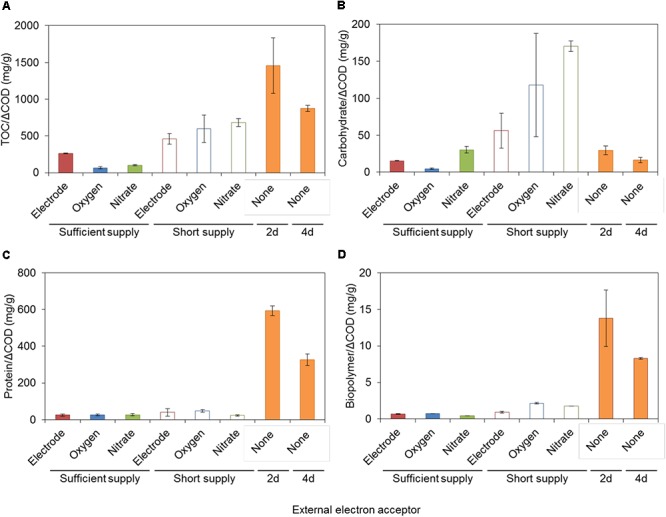
**(A)** TOC, **(B)** carbohydrate, **(C)** protein, and **(D)** biopolymer in SMP produced by strain S05 with different external electron acceptors. Statistical differences were estimated with *P*-value on the basis of two-sample *t*-test, which was summarized in Supplementary Table S2.

**Figure 4:**
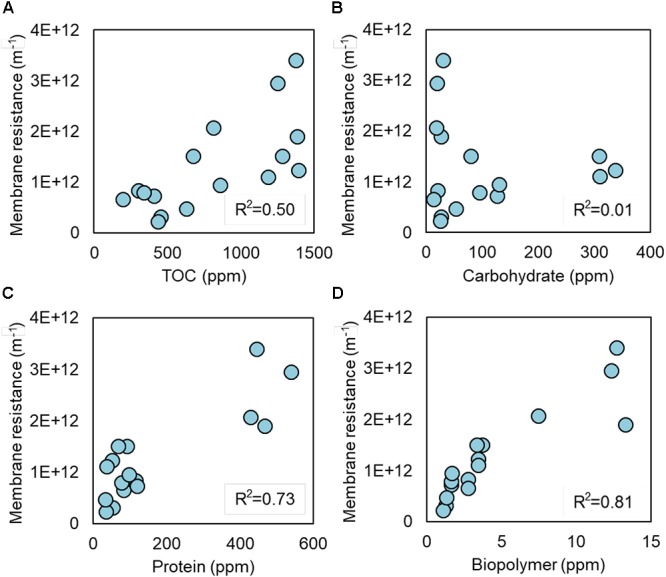
Linear correlation between membrane resistance and **(A)** TOC, **(B)** carbohydrate, **(C)** protein, and **(D)** biopolymer in SMP.

Soluble microbial products and EPS produced by S05 were characterized by measuring the concentrations of TOC, carbohydrate, protein, and biopolymer, which were highly dependent on the type of electron acceptors (Figures 3, 4, Supplementary Figure S6, and Supplementary Table S3). Specific protein and biopolymer production (mg-protein or biopolymer per g-COD removed) in SMP were significantly higher when cultured without any external electron acceptor (none) (*p* < 0.01 and *p* < 0.001, respectively) (Figures 3C,D). This trend was consistent with the results of membrane fouling potential (Figure 2). It should be noted that protein and biopolymer contents in SMP were positively correlated to membrane resistance (*R*^2^ = 0.73 and 0.81, respectively) (Figure 4).

**Figure 5:**
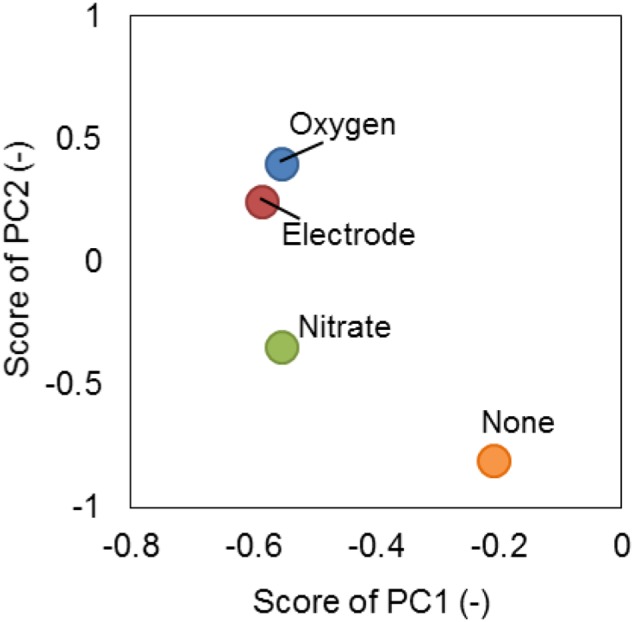
Principle compartment analysis (PCA) on the basis of FTIR spectrum of SMP produced by strain S05 cultured at sufficient supply of different external electron acceptors.

Moreover, SMP was characterized by PCA based on the FTIR spectrum (Figure 5). The pattern of the SMP spectrum was different from each other (Supplementary Figure S7). For example, the level of peak intensity of two spectra located at 1640 and 1560 cm^-1^, which represent the protein secondary structure known as amide I (N-C = O) and amide II (N = C-O), respectively (Pendashteh et al., 2011), was different from each other. This suggests that not only the total amount but also the composition of proteins in SMP were different under different electron acceptor conditions (Figures 3C, 5). The PCA plots of oxygen and anode electrode were rather close each other but apart from those of nitrate and none, suggesting that the composition of SMP was dependent on the type of external electron acceptors (Figure 5). In particular, the plot of SMP produced when cultured without any external electron acceptor (none) was far away from the other plots, indicating a significant composition difference.

### SEM Analysis

**Figure 6:**
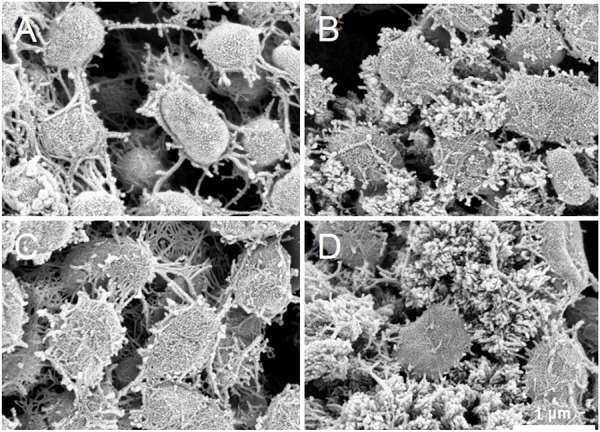
Scanning electron microscopy (SEM) images of biofilm established on porous carbon felt by strain S05 when cultured with **(A)** electrode, **(B)** oxygen, **(C)** nitrate, and **(D)** none as external electron acceptor, respectively (sufficient supply).

S05 cells cultured with different electron acceptors were observed with SEM to visualize EPS production (Figure 6). The anode biofilms formed on anode carbon felts under different electron acceptor conditions were observed although most of S05 was presented as planktonic cells in suspension regardless of the type of electron acceptor (Supplementary Figure S2). Under all the culture conditions, S05 produced and accumulated EPS around cells, but the appearance and amount of EPS were different. For example, the most EPS were found without any external electron acceptor (none) and the least EPS was found under anodic respiration condition. Furthermore, filamentous EPS interconnected cells were particularly characteristic when cultured with anode electrode (Figure 6A), whereas the cells cultured without any external electron acceptor were covered with cauliflower-like EPS (Figure 6D).

## Discussion

The effects of different external electron acceptors (anode electrode, oxygen, and nitrate) on the fouling potential of *K. quasipneumoniae* strain S05, an exoelectrogenic FCB, were investigated. The fouling potential was high with following order; no external electron acceptor (none, i.e., fermentation), nitrate, oxygen and anode electrode. Intrudingly, the fouling potential was lowest under anodic respiration condition and highest without any external electron acceptor. These results suggest the potential application of integrated MFC and MBR systems for wastewater treatment, in which aeration (oxygen respiration) could be replaced by anode electrodes (anodic respiration).

*Klebsiella quasipneumoniae* strain S05 is a facultative anaerobe and known to be capable of generating electricity and cause severe membrane fouling (Zhang et al., 2008; Xia et al., 2010; Kumar et al., 2015; Kim et al., 2016; Ishizaki et al., 2016a). In addition, *Klebsiella* species has been frequently detected in MFCs, MBRs, and integrated MFC and MBR systems (Yu et al., 2012; Jia et al., 2013; Khan et al., 2013; Ishizaki et al., 2016b; Win et al., 2016). In our previous study, the genus *Klebsiella* dominated more than the genus *Geobacter* in ML on the basis of 16S rRNA gene sequence (in a range of 0.25–0.57% vs. 0.003–0.05% of the total reads analyzed by next-generation sequencing, respectively) whereas the genus *Geobacter* were more dominantly found in anodic biofilms (in a range of 0.10–1.22% vs. 1.74–49.2% of the total reads, respectively) in the integrated MFC-MBR system (Ishizaki et al., 2016b). Furthermore, S05 had more severe membrane fouling potential than *G. sulfurreducens* strain PCA when cultured with anode electrode [7.1 ± 2.3 vs. 0.56 ± 0.03 (10^11^ m^-1^)] (Figure 2) (Ishizaki et al., 2016b). We also previously reported that introduction of anodic respiration could mitigate membrane fouling of mixed-population MBR due to reduction of biopolymer production (Ishizaki et al., 2016b) and addition of S05 to activated sludge significantly enhanced membrane fouling (Ishizaki et al., 2017). In order to verify the effect of anodic respiration on membrane fouling caused by exoelectrogenic FCB, membrane fouling potential of a pure-cultured S05 was determined under different electron acceptor conditions and correlated to SMP and EPS production and composition in this study.

*Klebsiella* species have a potential to use various types of external electron acceptors (i.e., respiration modes), under which microbial activity and metabolism change drastically. For example, *Klebsiella* sp. strain JHW3 oxidized more As (III) to As (V) when cultured with oxygen than with nitrate or solid-state anode electrode as the sole external electron acceptor (Nguyen et al., 2017). A recombinant *K. pneumoniae* L17 overexpressing aldehyde dehydrogenase (AldH) produced 1.7 time higher 3-hydropropionaic acid (3-HP) from glycerol when cultured with anode electrode applied a potential at +0.5 V (vs. Ag/AgCl) than without applied potential (Kim et al., 2017). This study clearly demonstrated that the yield and pathway of metabolites production was enhanced by controlling the extracellular redox states in a bioelectrochemical system (BES). It has been reported that the extracellular redox states (i.e., the availability and type of external electron acceptors) has considerable influences on intracellular redox balance (i.e., the ratio of NADH/NAD^+^), which is closely linked to regulation of microbial metabolisms (de Graef et al., 1999; Chen et al., 2011; Liu et al., 2013). Taken together, the different electron acceptors used in the present study have different reduction potentials (*E*_0_′, volt) and thus would influence the intracellular redox states (NADH/NAD^+^) and consequently the yield and types of metabolites production. Further study is required to verify this hypothesis.

Soluble microbial products can be divided into biomass (decay) associated products (BAP) and (substrate) utilization associated products (UAP) (Ni et al., 2011). In the absence or shortage of external electron acceptors, more BAP are expected to be generated due to biomass decay, which mostly composed of high molecular weight refractory organics including bacterial cells and/or EPS debris and biopolymer (Jiang et al., 2010; Yang et al., 2016). In the present study, the biopolymer associated with BAP could have been produced to some extent in addition to UAP associated one under no and/or short supply conditions, because biomass decay or cell lysis is expected to more frequently occur during fermentation (Newton et al., 2016). However, BAP and UAP could not be quantified separately in the present study, which needs to be addressed in the future.

The biopolymer derived from UAP have shown the higher fouling potential as compared with the one from BAP (Jiang et al., 2010). In addition, the biopolymer with high molecular weight (>1,000 kDa) have higher fouling potential than that with low molecular weight (<100 kDa) (Kimura et al., 2018). Microbial SMP consists mostly of carbohydrate, protein, and biopolymer (Figure 3), and cell debris such as outer membrane proteins were also known to cause severe membrane fouling (Miyoshi et al., 2012; Gao et al., 2013; Sun et al., 2014; Zhou et al., 2015). This study revealed that protein and biopolymer contents in SMP were highly correlated with the membrane fouling potential (*R*^2^ = 0.73 and 0.81, respectively). Taken together, the quantity, molecular size and compositions of SMP, especially biopolymer and proteins, are important factors determining membrane fouling, which also needs to be studied in the future.

To date, a variety of exoelectrogenic bacteria and FCB have been identified (Ishii et al., 2008; Logan, 2009; Torres et al., 2009; Kumar et al., 2015), only one isolated strain was investigated as a model microorganism in this study. Thus, identification and characterization of other exoelectrogenic FCB are necessary to understand whether these findings are S05 strain specific or more general characteristic of exoelectrogenic FCB. In addition, microbial and/or electrochemical mechanisms of membrane fouling mitigation under anodic respiration condition must be further investigated based on electrochemical analyses and molecular approach (i.e., transcriptome analysis) in the future (Malaeb et al., 2013b).

According to the results of the present study showing that the fouling potential was highest under no external electron acceptor condition (Figure 2), MBR must be designed and operated effectively without electron acceptor-limited spaces inside the reactor, in which fermentation and anaerobic biomass degradation occur (Jin et al., 2006; Lin et al., 2013; Ma et al., 2015; Ishizaki et al., 2016b). Instead of applying energy intensive aeration, anode electrodes could be installed to prevent the shortage of electron acceptor in the reactor. Therefore, the integration of MFC and MBR is expected to be feasible now as energy-saving wastewater treatment. Membrane fouling can be further mitigated by electrochemically controlling the intracellular and extracellular redox states, which derive target microbial metabolisms to suppress the production of foulants. Further studies are necessary to optimize the operational conditions including electrochemical potentials to regulate the metabolisms and to optimize the reactor configuration and arrangement of anode electrodes in the integrated MFC and MBR system.

## Conclusion

The effect of different external electron acceptors (electrode, oxygen, and nitrate) on membrane fouling potential and the bacterial secretion (SMP and EPS) by *K. quasipneumoniae* strain S05, an exoelectrogenic FCB, were investigated. Most of S05 grew as planktonic cells in suspension regardless of the type of electron acceptor. The fouling potential was high with following order; no external electron acceptor (none, i.e., fermentation), nitrate, oxygen and anode electrode (anodic respiration condition). SMP and EPS composition was dependent on the availability and the type of electron acceptor. The highest production of biopolymer and protein, the main foulants, was observed in the absence of external electron acceptor. Enhancement of anodic respiration by regulating anodic electrode potential was effective to mitigate the foulant production and consequently membrane fouling. In addition, the prevention of shortage of external electron acceptor in MBR is essential to reduce the membrane fouling.

## Author Contributions

SI and SO designed research. SI, RIP, HM, and YN performed research. SI and SO wrote the manuscript.

## Conflict of Interest Statement

The authors declare that the research was conducted in the absence of any commercial or financial relationships that could be construed as a potential conflict of interest.
