# Supplementary material for: Membrane Fouling Potentials of an Exoelectrogenic Fouling-Causing Bacterium Cultured With Different External Electron Acceptors
Source: Front Microbiol. 2019 Jan 14;9:3284. doi: 10.3389/fmicb.2018.03284 (PMC6340052; doi:10.3389/fmicb.2018.03284)

**Supplemental Information**

**Membrane fouling potentials of an exoelectrogenic fouling-causing bacterium cultured with different external electron acceptors**

So Ishizaki^1^, Papry Rimana Islam^1^, Hiroshi Miyake^1^, Yuko Narita^1^, and Satoshi Okabe^1^*

**Supporting information**

11 pages

7 Figures

3 Tables

^1^Division of Environmental Engineering, Faculty of Engineering, Hokkaido University, North 13, West 8, Kita-ku, Sapporo, Hokkaido 060-8628, Japan

*Corresponding Author

Satoshi Okabe

[sokabe@eng.hokudai.ac.jp](mailto:sokabe@eng.hokudai.ac.jp), Telephone/Fax: +81-11-706-6266


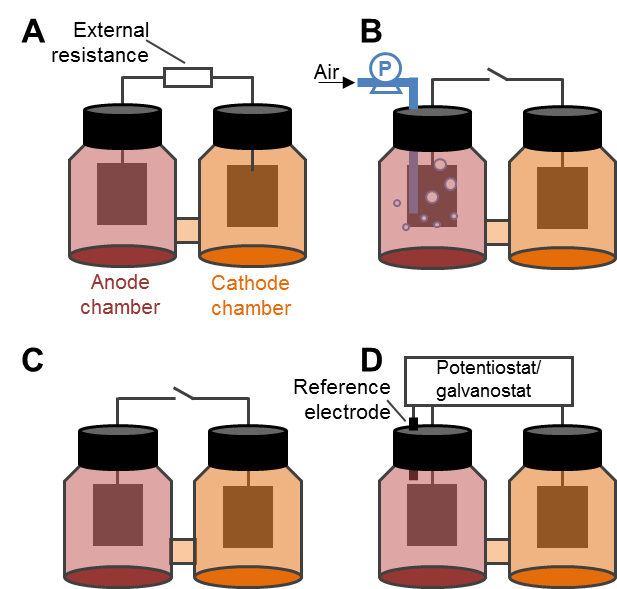


**Figure S1.** Illustration of double-chamber reactors operated with different electron acceptors; (**A**) electrode at short supply, (**B**) oxygen, (**C**) nitrate and none, and (**D**) electrode at sufficient supply. The reactor A and D, and B and C were operated as closed-circuit MFC and open-circuit MFC, respectively. The reactor D was equipped with a potentiostat/galvanostat to regulate anodic electrode potential. In this reactor, anode and cathode electrode acted as working and counter electrode, respectively.

**
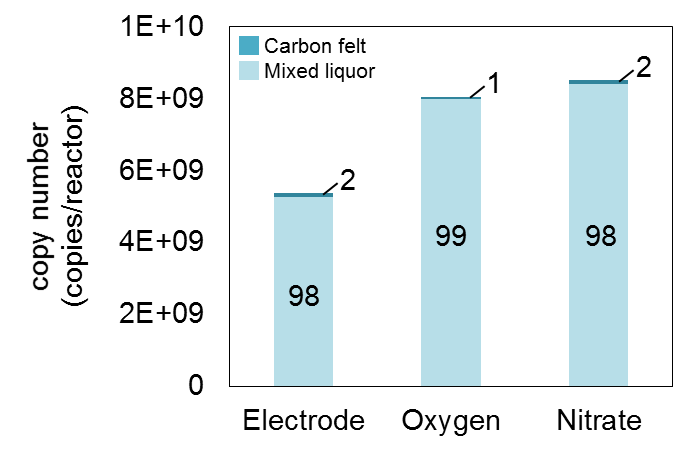
**

**Figure S2.** Abundances of 16S rRNA genes of strain S05 in mixed liquor and on carbon felt (electrode) in reactor when cultured with different electron acceptor at short supply. The numbers in each bar are the relative percentage, respectively.


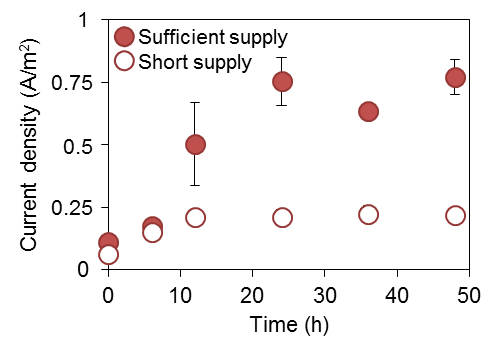


**Figure S3.** Time variation in electrical current generated by strain S05


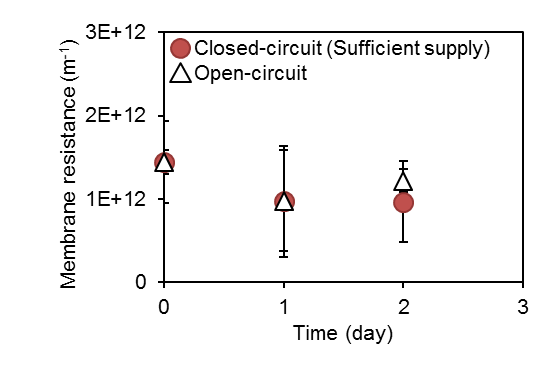


**Figure S4.** Abiotic effect of regulation of anodic electrode potential on fouling potential. Sterilized SMP of strain S05 was incubated in open-circuit and closed-circuit MFC reactor and subsequently the fouling potential was monitored for 2 days.


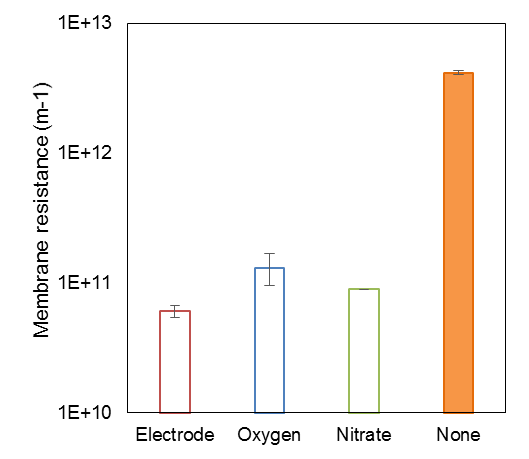


**Figure S5.** Fouling potential of S32 when cultured with different external electron acceptors at short supply.

.


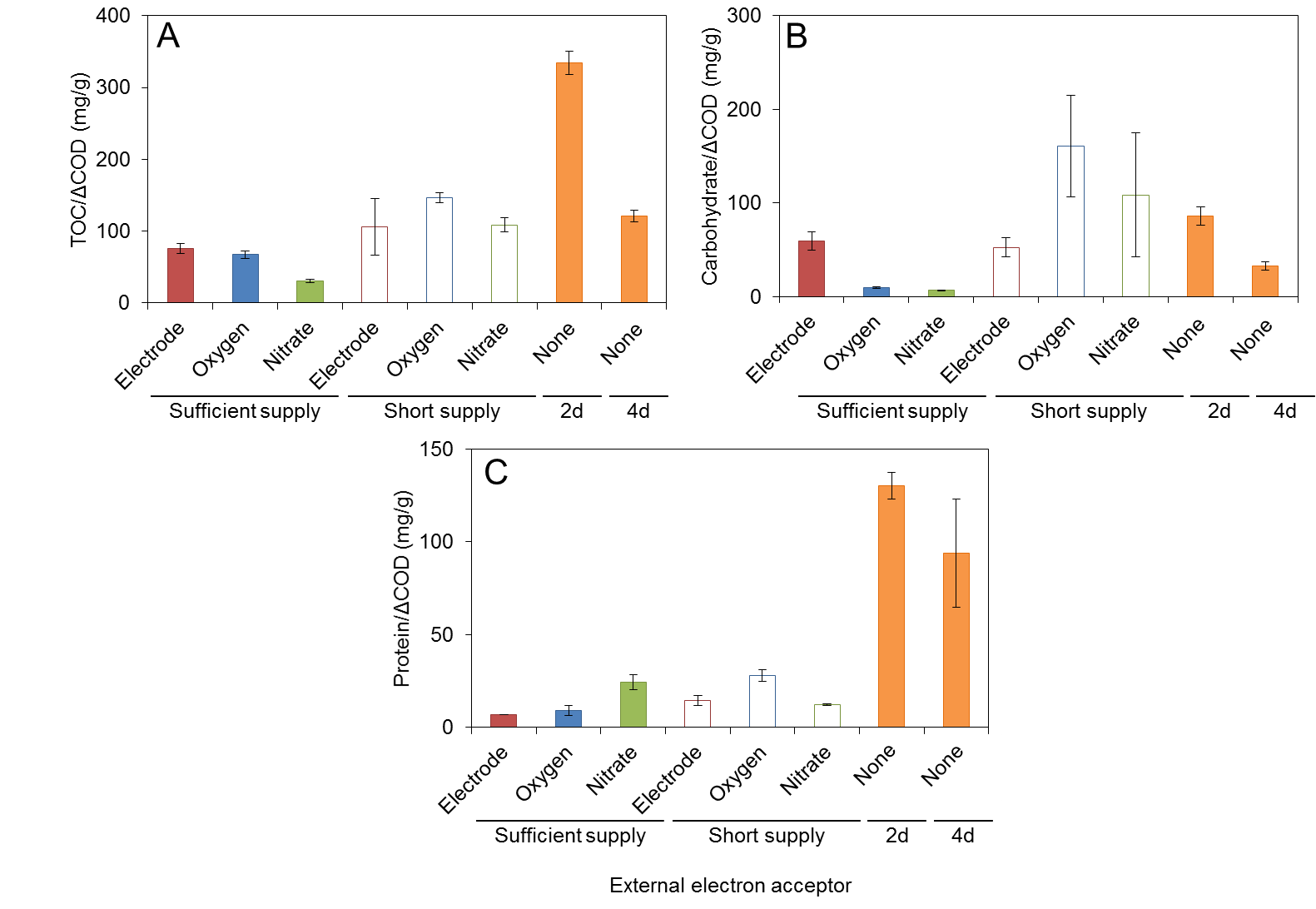


**Figure S6.** **A**) TOC, **B**) carbohydrate, and **C**) protein in EPS produced by strain S05 with different external electron acceptors. Statistical differences were estimated with *P*-value on the basis of two-sample *t*-test, which was summarized in **Table S3**.


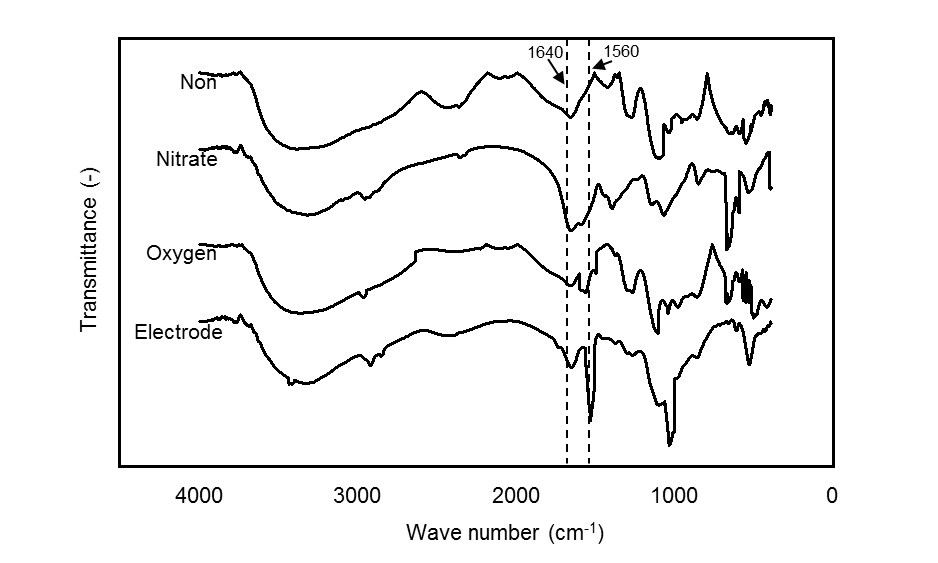


**Figure S7.** FTIR spectra of SMP produced by strain S05 cultured at sufficient supply of different external electron acceptors.

**Table S1.** Statistically significant difference between two groups in membrane fouling potential shown in **Fig. 2.** The difference was estimated with *P*-value on the basis of two-sample *t*-test: ***, *P*<0.001; **, *P*<0.01; *, *P*<0.05.


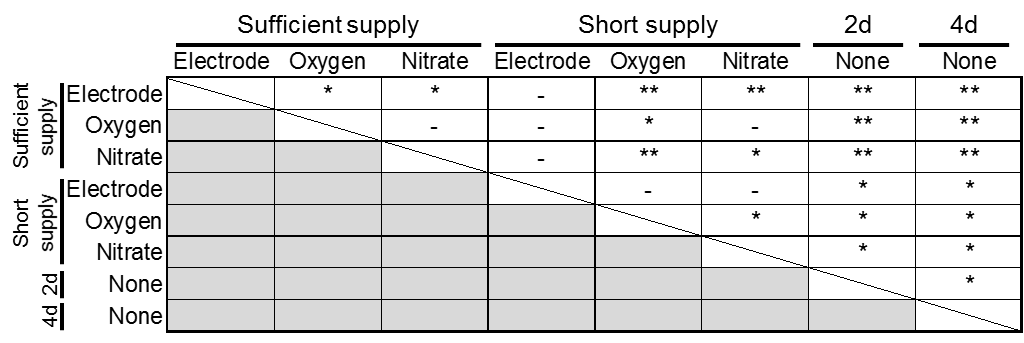


**Table S2.** Statistically significant difference between two groups in **A**) TOC, **B**), Carbohydrate, **C**) Protein, and **D**) Biopolymer in SMP shown in **Fig. 3.** The difference was estimated with *P*-value on the basis of two-sample *t*-test: ***, *P*<0.001; **, *P*<0.01; *, *P*<0.05.


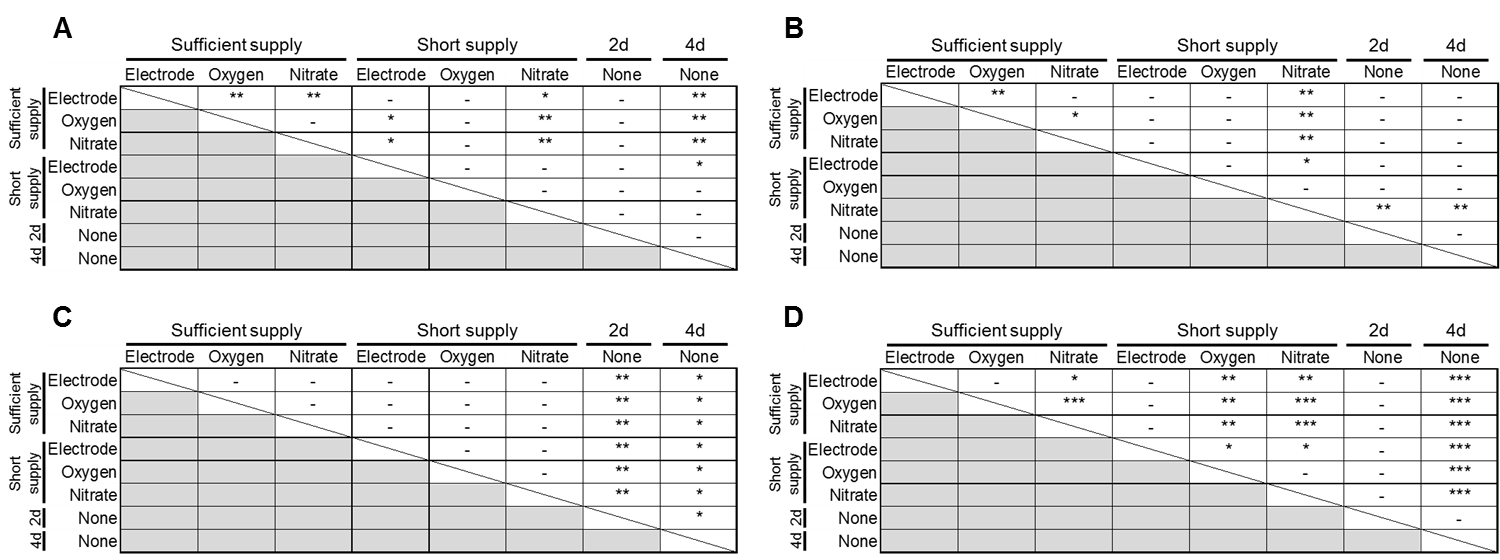


**Table S3.** Statistically significant difference between two groups in **A**) TOC, **B**), Carbohydrate, and **C**) Protein in EPS shown in **Fig. 3.** The difference was estimated with *P*-value on the basis of two-sample *t*-test: ***, *P*<0.001; **, *P*<0.01; *, *P*<0.05.


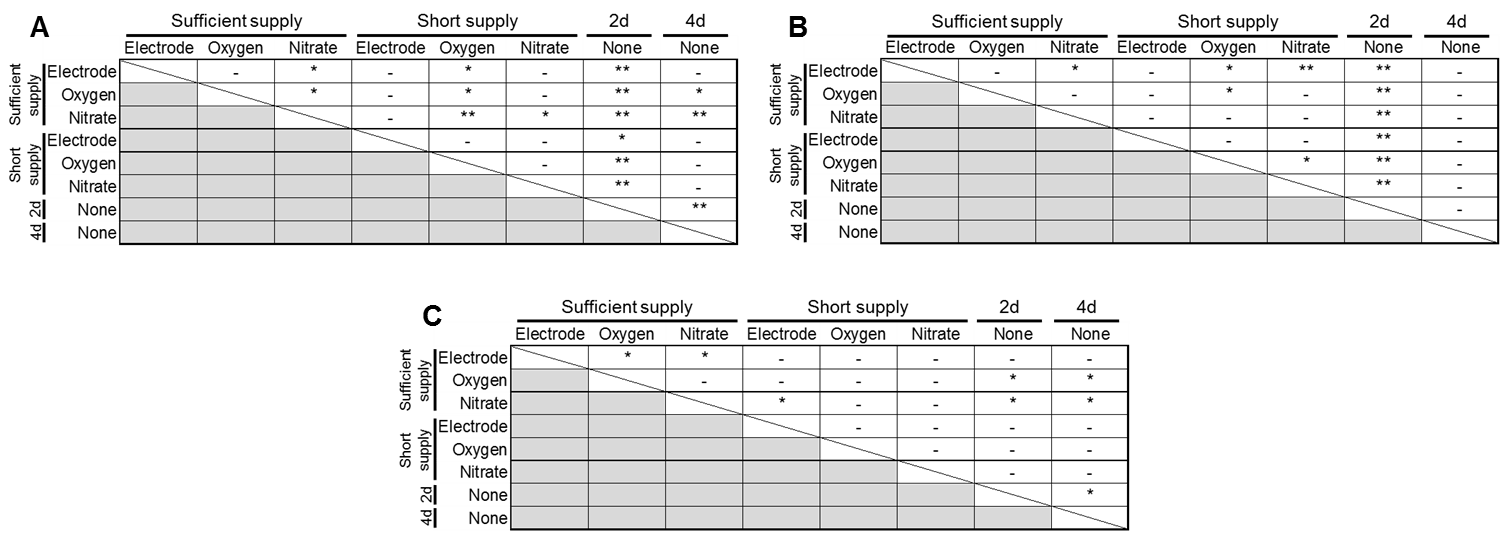

Supplement: Supplementary file 1 [file Data_Sheet_1.doc]
